# A Case of Evolving Hepatotoxicity Following Systemic Drug Reaction to Isoniazid–Rifapentine (3HP)

**DOI:** 10.1155/crhe/2508892

**Published:** 2025-11-24

**Authors:** Niels Vizgan, Jesse Martinez-Kratz, Chris LaBudde, Fredric Gordon

**Affiliations:** ^1^School of Medicine, Tufts University, Boston, Massachusetts, USA; ^2^Abdominal Transplant Institute, Tufts Medical Center, Boston, Massachusetts, USA

## Abstract

The 3-month, once-weekly regimen of isoniazid plus rifapentine (3HP) is widely used for latent tuberculosis infection (LTBI) because of its shorter duration and favorable adherence compared to isoniazid monotherapy. However, 3HP is associated with hepatotoxicity as well as systemic drug reactions (SDRs), characterized by rapid-onset flu-like symptoms, fever, myalgias, and rash, which may complicate therapy. We describe a healthy 26-year-old male diagnosed with latent tuberculosis who developed acute SDR symptoms accompanied by hepatotoxicity after his third dose of 3HP. The liver injury was initially cholestatic and evolved into a worsening hepatocellular pattern despite discontinuation of 3HP, with gradual normalization over 4 weeks. Autoimmune serologies were briefly positive but resolved spontaneously without intervention. This case illustrates the potential for SDRs with evolving liver injury during 3HP therapy and underscores the importance of early recognition of adverse effects and individualized management.

## 1. Introduction

Latent tuberculosis infection (LTBI) is commonly treated to prevent progression to active disease, particularly in individuals with occupational or healthcare-related exposure risk [1, 2]. The once-weekly combination of rifapentine and isoniazid (3HP) for 12 weeks has emerged as a preferred LTBI regimen due to its shorter duration and higher adherence rates compared to traditional 9-month daily isoniazid therapy (9H) [3–5]. While generally well tolerated, 3HP has been associated with hepatotoxicity and systemic drug reactions (SDRs), a distinct syndrome characterized by flu-like symptoms, fever, myalgias, and rash, which may mimic or coexist with drug-induced liver injury (DILI) [3, 6–8].

Rifapentine, a rifamycin similar to rifampin, inhibits bacterial RNA polymerase. Isoniazid inhibits mycolic acid synthesis after activation by the bacterial KatG enzyme [9, 10] and has been linked to idiosyncratic hepatotoxicity and, rarely, hypersensitivity reactions [11–13]. Compared to daily isoniazid, 3HP has been associated with earlier onset of hepatotoxicity and a higher frequency of systemic flu-like syndromes [6, 7].

We present a case of a 26-year-old male with LTBI treated with 3HP who developed a SDR (fever, myalgias, and rash) accompanied by hepatotoxicity that initially worsened after discontinuation before ultimately resolving, underscoring the diagnostic and management challenges of 3HP therapy. This case emphasizes the need for vigilant monitoring of liver chemistries and systemic symptoms, as well as patient education on prompt reporting of adverse effects.

## 2. Case

A 26-year-old male with a past surgical history of gastric bypass and a history of asymptomatic sinus bradycardia (resting heart rate in the 40 s, attributed to athletic conditioning) was diagnosed with LTBI on an annual QuantiFERON-TB Gold test required for health professional school. No prior tuberculosis exposures were known. Due to ongoing clinical training and patient-facing responsibilities, he elected to begin weekly 3HP therapy, dosed as isoniazid 900 mg plus rifapentine 900 mg weekly, with concurrent weekly 50 mg of pyridoxine (vitamin B6) supplementation. Therapy was not directly observed due to scheduling constraints.

**Figure 1:**
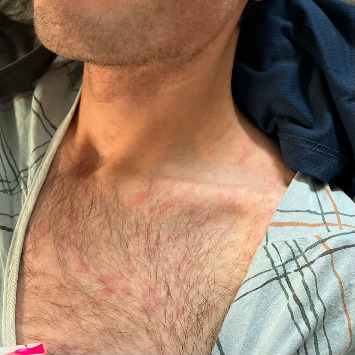
Dermatologic findings notable for diffuse, nontender, nonmigratory, nonpruritic, and erythematous macules sparing mucous membranes and the palms and soles.

Three hours after his third dose (Day 15 after therapy initiation), he developed fevers, chills, myalgias, and a diffuse erythematous rash on the arms and back. The prior doses did not elicit any symptoms. He denied syncope, lightheadedness, alcohol or substance use, or concurrent medications. He presented to the emergency department where on arrival, his temperature was 103.1°F, and his heart rate was 98–107 bpm, markedly elevated relative to his baseline bradycardia. Blood pressure was stable with no orthostatic changes. Physical exam revealed a diffuse, nonpruritic, nontender erythematous rash (Figure 1) sparing the palms and soles. Laboratory testing showed AST 149 U/L, ALT 94 U/L, alkaline phosphatase 135 U/L, total bilirubin 1.2 mg/dL, direct bilirubin 0.7 mg/dL (normal: 0–0.3), platelets 117 × 10^9/L (normal: 150–400), and GGT 61 IU/L (normal: 0–65). Prothrombin time (PT) was elevated at 15.9 s (normal: 10–13) and INR 1.38 (normal: 0.8–1.1). C-Reactive protein (CRP) was elevated at 61 mg/L, and erythrocyte sedimentation rate (ESR) was normal at 12 mm/h. HIV and hepatitis A, B, and C testing were negative. He was found to have normal ceruloplasmin and normal α-1 antitrypsin. Chest imaging revealed no active tuberculosis, and a nonobstructive 2.7-cm gallstone was noted on abdominal ultrasound without evidence of cholecystitis. The patient was admitted and fluid resuscitated, and 3HP therapy was discontinued. He was discharged after a liver chemistry panel, and his symptoms were noted to improve; he was instructed to repeat labs as an outpatient.

**Figure 2:**
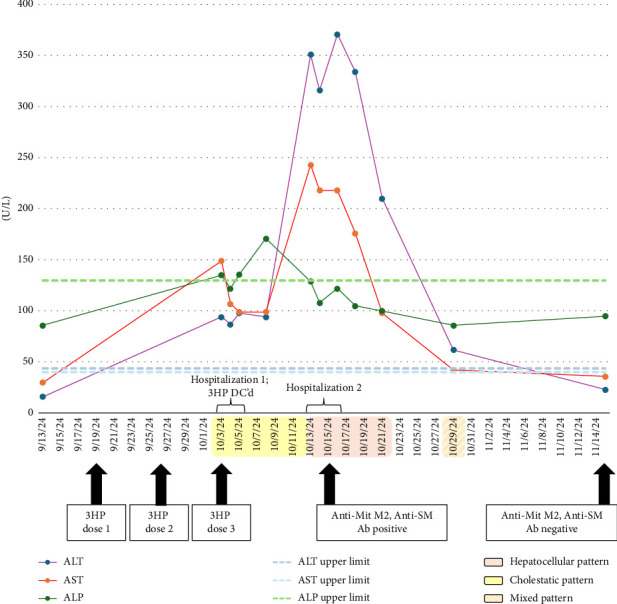
Line plot connecting discrete liver chemistries drawn prior to and following initiation of weekly isoniazid plus rifapentine (3HP). Alanine aminotransferase (ALT, purple), aspartate aminotransferase (AST, red), and alkaline phosphatase (ALP, green) are plotted over time with upper limits of normal shown as dashed lines. After the third 3HP dose (Day 15), the patient developed systemic symptoms and was hospitalized with an initial cholestatic pattern (yellow shading). Despite discontinuation of therapy, liver injury progressed to a hepatocellular pattern (orange shading) with marked aminotransferase elevation and subsequent hospitalization. During recovery, a brief mixed pattern was observed (striped shading). Arrows denote timing of 3HP dosing, hospitalization, and transient AMA/ASMA positivity followed by spontaneous resolution.

Eight days later, the patient was asymptomatic, but outpatient labs demonstrated worsening liver chemistries (AST 198 U/L, ALT 308 U/L, alkaline phosphatase 99 U/L, and total bilirubin 1.2 mg/dL), and he was readmitted under observation. They continued to worsen in patient with AST 243 U/L, ALT 351 U/L, alkaline phosphatase 129 U/L (Figure 2), and total bilirubin 1.1 mg/dL. PT-INR was normal. Magnetic resonance cholangiopancreatography (MRCP) was performed to rule out Mirizzi syndrome and confirmed the gallstone presence without evidence of biliary obstruction. For concerns for a potential underlying autoimmune etiology worsened by 3HP, autoimmune studies were ordered. These revealed elevated IgG, 2180 mg/dL (range: 700–1600); elevated IgA, 426.0 mg/dL (range: 70.0–400.0); positive antismooth muscle antibody (ASMA), 1:40; positive antimitochondrial antibody (AMA) M2 phenotype, 120.3 units; and negative ANA. The patient remained clinically stable. Liver biopsy was deferred in favor of discharge with close outpatient monitoring. Serial outpatient monitoring demonstrated gradual improvement, with normalization of liver chemistries by Day 25 postdiscontinuation. Hepatology continued to follow until Day 43, when repeat serologies were negative for ASMA and AMA. Infectious disease elected to defer all further LTBI therapy in this patient. Isoniazid was documented as an allergy in his electronic medical record.

## 3. Discussion

The timing and constellation of symptoms in this patient are consistent with a SDR, a distinct syndrome characterized by flu-like symptoms such as fever, myalgias, and rash, which is a known potential side effect of 3HP. In the pivotal PREVENT TB trial, SDRs occurred in 3.5% of 3HP recipients, as compared to 0.4% of recipients who had received the traditional 9H. They were characterized as flu-like (63%) or cutaneous (0.6%), typically appeared after the third dose, occurred ∼4 h postdose, and resolved within ∼24 h [6]. In a multicenter study of ∼3300 patients, 36% of 3HP recipients experienced at least one adverse event, with fever or chills in ∼12%–15%, rash in ∼6%–8%, and other constitutional complaints in a substantial minority [14]. Despite this, > 85% completed therapy, underscoring that most events are mild and self-limited. Together, these data highlight that systemic reactions with 3HP are relatively common, even without hepatotoxicity, and require anticipatory counseling and monitoring in clinical practice.

Both components of 3HP have idiosyncratic hepatotoxicity profiles, characterized by variable onset, lack of clear dose dependence, and immune-mediated features. Rifamycins such as rifapentine are most often associated with cholestatic or mixed liver injury, typically occurring early in treatment [11]. By contrast, isoniazid-related liver injury is usually hepatocellular and more delayed, with onset often after months of therapy [11–13]. Pharmacogenomic data suggest that isoniazid slow acetylator genotypes (*NAT2* variants), and higher serum INH concentrations may predispose individuals to both hepatotoxicity and SDRs during 3HP therapy [15]. Data from rifampin monotherapy (4R) suggest that rifamycins as a class may be less hepatotoxic than isoniazid, with a randomized trial showing fewer severe hepatic events and better overall tolerability with 4R compared to 9H [16]. Compared with 9H, hepatotoxicity in 3HP tends to occur earlier, with a median onset of 22 days versus 101 days for INH alone [7]. Liver chemistry abnormalities in 3HP-related clinical cohorts are most often hepatocellular, sometimes with transient cholestasis [6, 7].

The evolution of liver chemistries in this case illustrates these complexities. The initial pattern was predominantly cholestatic, with modest aminotransferase elevations and a peak in alkaline phosphatase; however, after discontinuation of 3HP, the profile shifted to hepatocellular, with a marked rise in ALT and AST (ALT > AST), while ALP declined (Figure 2). This biphasic course highlights how 3HP can produce evolving patterns that do not align neatly with the classical signatures of either rifamycins or isoniazid alone. Indeed, the evolving pattern helped to distinguish the liver injury from primary autoimmune or cholestatic disease and demonstrated the diagnostic value of following the liver chemistry trajectory in suspected DILI.

Ancillary laboratory data align with a SDR rather than primary autoimmune pathology. CRP elevation and transient thrombocytopenia can occur with SDRs, in contrast to the abrupt, severe thrombocytopenia that characterizes immune-mediated rifamycin reactions [17]. The transient AMA and ASMA positivity in this case likewise resolved spontaneously and is best interpreted as secondary to hepatocellular stress, rather than as evidence of primary autoimmune disease such as primary biliary cholangitis or autoimmune hepatitis [18–20].

This case illustrates that 3HP-related hepatotoxicity may emerge early, can persist even after discontinuation, and may be accompanied by transient autoantibody positivity that should be interpreted in context rather than as evidence of chronic autoimmune disease. It underscores the importance of close clinical and laboratory monitoring, patient education about systemic symptoms, and adherence to ATS/CDC/IDSA [3] and NTCA/NSTC [4] guideline recommendations, which outline specific thresholds for discontinuing therapy. With early recognition and supportive management, patients typically achieve full recovery.

## Practical Application

Early recognition of hepatotoxicity and systemic symptoms is critical. Liver injury can worsen even after drug discontinuation.

Patient education is essential: Patients should be instructed to pause therapy and seek medical attention promptly if symptoms such as fever, rash, or malaise occur.

Treatment selection should weigh risk-benefit: Isoniazid monotherapy (9H) carries the highest hepatotoxicity risk, 4R has the lowest adverse event rate, whereas 3HP offers shorter duration and higher adherence but a higher incidence of transient systemic reactions [3, 16, 21].
